# Nutational resonance modes in antiferromagnetic materials

**DOI:** 10.1038/s41598-025-08746-0

**Published:** 2025-07-01

**Authors:** David Angster, Tobias Dannegger, Julius Schlegel, Martin Evers, Ulrich Nowak

**Affiliations:** Fachbereich Physik, Universität Konstanz, D-78457 Konstanz, Germany

**Keywords:** Nutation, Antiferromagnetic Resonance, Spintronics, Spintronics, Magnetic properties and materials, Atomistic models

## Abstract

The Landau–Lifshitz–Gilbert (LLG) equation is well-established to describe the spin dynamics of magnetic materials. This first-order differential equation is based on the assumption that the spin angular momenta and corresponding magnetic moments are always parallel. While this assumption is largely unproblematic, both theoretical considerations and experimental results have indicated that the two may become separated on ultrafast timescales, giving rise to inertial dynamics along with a modified spin wave dispersion. Here, we apply linear spin wave theory to the inertial LLG equation to compute the eigenmodes of the altermagnetic materials SmErFeO_3_ and $\alpha$-Fe_2_O_3_. We find the largest influence of nutation on the magnetic resonances in the case of hematite, which exhibits both a sizeable shift of the resonance frequencies as compared to the inertia-free case and additional nutational resonances that are in a similar order of magnitude to the materials’ higher-frequency precessional exchange modes. While the realistic magnitude of the inertial parameter remains an open question, we hope that our quantitative analysis provides the starting point for further experimental investigations.

## Introduction

The common equation to describe atomistic magnetisation dynamics is the well-established Landau-Lifshitz-Gilbert (LLG) equation, accounting for both precession and relaxation phenomena^1–3^. It explains the precession of magnetic moments around an effective field and, in magnetically ordered systems, the appearance of spin waves or, in their quantised form, magnons.

On ultrashort timescales, the assumption that magnetic moment and angular momentum are strictly aligned, which is necessary to derive the LLG equation, is no longer valid. In analogy to a mechanical gyroscope, with the body axis corresponding to the magnetic moment, this leads to nutation, and the LLG equation has to be extended with an inertia term, which contains the second-order time derivative of the magnetic moment’s trajectory and yields the inertial Landau-Lifshitz-Gilbert (ILLG) equation^4,5^. In addition to this phenomenological derivation, the ILLG can also be obtained via first principles calculations and relativistic quantum theory^6–8^. It has been suggested that inertial properties of spin dynamics could be used to facilitate ultrafast magnetisation switching^9,10^.

**Fig. 1:**
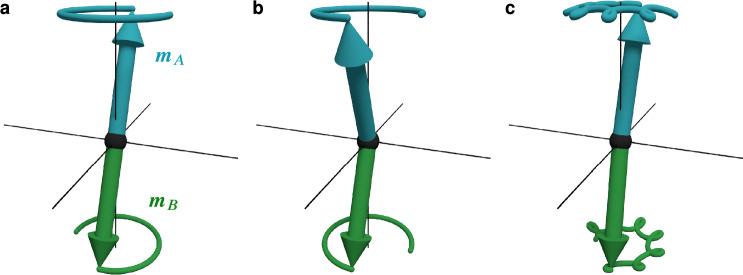
Sketch of precessional (**a**) and nutational (**b**) eigenmodes of an antiferromagnet with inertia. A superposition of both modes (**c**) leads to a nutating precession of the sublattice magnetisation vectors $\varvec{m}_{A,B}$. Because this is an antiferromagnet, there also exists another set of modes with the opposite sense of rotation.

In terms of (anti)ferromagnetic resonance, the inertial term leads to additional high-frequency resonance modes. As an example, Fig. 1 shows the well-known precessional resonance mode of a two-sublattice antiferromagnet (Fig. 1a) together with the nutational resonance mode (Fig. 1b), which is expected to have a much larger frequency and opposite helicity. If we superimpose both modes, we get a combined excitation where the sublattice magnetisation vectors trace out a path reminiscent of mechanical nutating spinning tops (Fig. 1c).

The existing theoretical work on magnetic nutation and on its various signatures focuses on rather general models which are not material-specific. The experimental verification is still in its infancy^9,11–13^, mainly because the most obvious signatures of nutation, the nutational resonances, have much higher frequencies than their known precessional counterparts. Hence, theoretical investigations of nutational spin dynamics in real materials are necessary to provide theoretical predictions that are comparable to experiments.

In this work, we extend the concept of linear spin-wave theory from the LLG to the ILLG to analytically calculate both the precessional and nutational eigenmodes of a general extended Heisenberg model. We apply this theory first to simple ferromagnetic and antiferromagnetic toy models before proceeding to real materials that are of current interest in antiferromagnetic spintronics research. In particular, we use quantitatively accurate models of a rare-earth orthoferrite, ${\hbox {Sm}_{0.7}\hbox {Er}_{0.3}\hbox {FeO}_{3}}$^14^ and hematite, ${\alpha -\hbox {Fe}_2\hbox {O}_3}$^15^ (both also candidates for altermagnetism^16^). We determine the frequencies and lifetimes of the resonance modes (with wavevector $\varvec{k} = 0$), as a function of the nutation parameter $\eta$. We also calculate the precise dynamics of the sublattice magnetisation vectors and the resulting net magnetisation of the total system, to predict how each resonance mode is reflected in measurable components of the magnetisation vector. With our work, we intend to stake out the nature and realistic range of inertial effects that can be expected in real-life antiferromagnetic resonance measurements and thereby provide a frame of reference for future experimental investigations. We focus here on the resonance modes because they are the most straightforward to measure and comparatively simple to understand theoretically, but our theory could in future also be extended to finite $\varvec{k}$-vectors.

For classical normalised magnetic moment vectors $\varvec{S}^i$ experiencing an effective field $\varvec{H}^i = -\partial \mathcal {H}/\partial \varvec{S}^i$ (for a given Hamilton function $\mathcal {H}$), the ILLG equation reads^4,5,7^

1 $$\begin{aligned} \frac{\mathrm d\varvec{S}^i}{\mathrm dt} = -\frac{\gamma }{\mu _S^i}\varvec{S}^i\times \varvec{H}^i + \alpha \varvec{S}^i\times \frac{\mathrm d\varvec{S}^i}{\mathrm dt} + \eta \varvec{S}^i\times \frac{\mathrm d^2\varvec{S}^i}{\mathrm dt^2}, \end{aligned}$$

where $\eta$ is the inertial parameter, expected to range from about 1 to 100fs^4,5^, $\alpha$ is the Gilbert damping parameter and $\gamma$ is the absolute value of the gyromagnetic ratio. By forming the cross product of $\varvec{S}^i$ and the ILLG equation (1) and then solving for the second-order time derivative, we obtain the explicit form of the ILLG equation^17,18^,

2 $$\begin{aligned} \frac{\mathrm d^2\varvec{S}^i}{\mathrm dt^2} = -\frac{\gamma }{\mu _S^i\eta }\varvec{S}^i\times \left( \varvec{S}^i\times \varvec{H}^i\right) - \frac{\alpha }{\eta }\frac{\mathrm d\varvec{S}^i}{\mathrm dt} - \frac{1}{\eta }\varvec{S}^i\times \frac{\mathrm d\varvec{S}^i}{\mathrm dt} - \varvec{S}^i\left( \frac{\mathrm d\varvec{S}^i}{\mathrm dt}\right) ^2, \end{aligned}$$

which will be used for the following calculations.

The materials we investigate here can all be described by an extended semi-classical Heisenberg model with a Hamiltonian of the form

3 $$\begin{aligned} \mathcal {H} = -\frac{1}{2}\sum _{i,j} (\varvec{S}^i)^T\mathfrak {J}^{ij}\varvec{S}^j - \sum _i (\varvec{S}^i)^T\mathfrak {K}^i\varvec{S}^i - \sum _i\sum _{\beta \eta \kappa \lambda } \mathfrak {L}^i_{\beta \eta \kappa \lambda } S^i_\beta S^i_\eta S^i_\kappa S^i_\lambda - \sum _i \mu ^i_S\varvec{B}\cdot \varvec{S}^i, \end{aligned}$$

where the $\varvec{S}^i$ are unit vectors representing the normalised magnetic moment of each magnetic atom *i*. The interatomic interaction between spins on lattice sites *i* and *j* is modelled by the exchange tensor $\mathfrak {J}^{ij}$, which, in addition to the isotropic exchange, may include the antisymmetric Dzyaloshinskii–Moriya interaction (DMI), as well as two-ion anisotropies, including dipole-dipole interactions. The magnetocrystalline on-site anisotropy of atom *i* is expanded in symmetric tensors of second order ($\mathfrak {K}^i$) and fourth order ($\mathfrak {L}^i$). Here, the Greek indices $\beta , \eta , \kappa$ and $\lambda$ denote the cartesian coordinates of the spin vectors. Higher-order anisotropy tensors could also be added but are not necessary in our case. The last term is the Zeeman energy of the atomic magnetic moment $\mu ^i_S \varvec{S}^i$ ($\mu ^i_S$ is the magnitude of the magnetic moment and $\varvec{S}^i$ is the unit vector specifying its direction) within an external magnetic field $\varvec{B}$.

For a system whose ground state is collinear with the *z* axis, and in the case of small excitations, i.e. $|S_x,S_y|\ll |S_z|\approx 1$, the resulting effective field for a single spin on lattice site *i* can be linearised with respect to $S_x$ and $S_y$ and inserted into the explicit ILLG equation (2). The result may then be linearised again, producing coupled differential equations for the time evolution of the spins at different lattice sites. Since we are only interested in the resonance modes of the system, we can finally average over all spins in each sublattice to obtain a 4*N*-dimensional (*N* being the number of sublattices of the system) linear homogeneous system of ordinary differential equations that describes the inertial spin dynamics and takes the form

4 $$\begin{aligned} \frac{\mathrm d}{\mathrm dt} \hat{\mathcal {S}} = M \hat{\mathcal {S}} \end{aligned}$$

with the vector of spin wave amplitudes $\hat{\mathcal {S}} := \left( {^\textrm{A}\!}\hat{S}_x, {^\textrm{A}\!}\hat{S}_y, {^\textrm{A}\!}\dot{\hat{S}}_x, {^\textrm{A}\!}\dot{\hat{S}}_y, {^\textrm{B}\!}\hat{S}_x, \dots \right) ^T$, where the matrix *M* contains the parameters of the Hamiltonian in Eq. (3). Assuming $\lambda _j$ and $\varvec{v}_j$ ($j \in \{1,\ldots 4N\}$) are the eigenvalues and eigenvectors of the coefficient matrix *M*, the spin dynamics of the resonance modes are determined by the fundamental solutions to Eq. (4), which take the form $\hat{\mathcal {S}}_j(t) := \varvec{v}_j \exp (\lambda _j\cdot t)$. Since the matrix *M* is real-valued, the eigenvalues and eigenvectors come in pairs of complex conjugates. Thus, the 2*N* unique real solutions to Eq. (4) can be constructed via

5 $$\begin{aligned} \hat{\mathcal {S}}_{j,\text {real}}(t) := \varvec{v}_j \exp (\lambda _j t) + \bar{\varvec{v}}_j \exp (\bar{\lambda }_j t) \propto \text {Re}\left[ \varvec{v}_j \exp (\lambda _j t)\right] , \quad j=1,...,2N. \end{aligned}$$

Based on the definition of *M*, the components of $\hat{\mathcal {S}}_{j,\text {real}}$ correspond to the *x*- or *y*-components of one of the sublattice magnetisation vectors in mode *j*. The frequencies and lifetimes of these 2*N* unique resonance modes are given by $\omega _j = \pm \text {Im}(\lambda _j)$ and $\tau _j = -1/\text {Re}(\lambda _j)$.

In the case of a system with a non-collinear ground state, the coordinate system can be transformed locally with sublattice-specific rotational transformation such that the ground state configuration is parallel to the *z*-axis. This leaves the above procedure invariant, which can thus be applied in the same manner as before to calculate the resonance frequencies, lifetimes and spin dynamics. The detailed procedure can be found in the methods section.

## Results

### Nutation in ferro- and antiferromagnetic toy models

#### Generic ferromagnet

First, we will briefly introduce fundamental aspects of the inertial spin dynamics in a simple ferromagnetic toy model, which includes second- and fourth-order uniaxial on-site anisotropy *K* and *L*, isotropic exchange $J_0$, and two-ion anisotropy $\kappa$ in the form $J_z=J_0+2\kappa$ and $J_{\perp } := J_x = J_y = J_0-\kappa$. In this case, the linearised effective field on the lattice site *l* reads

6 $$\begin{aligned} \varvec{H}^l \approx \biggl (\sum _j J_{\perp } S^j_x \hat{\varvec{e}}_x + J_{\perp } S^j_y \hat{\varvec{e}}_y + J_z S^j_z \hat{\varvec{e}}_z \biggr ) + \biggl (2KS^l_z + L (S^l_z)^3 + \mu _s B\biggr )\hat{\varvec{e}}_z. \end{aligned}$$

In the absence of inertial effects, the resonance frequency and lifetime of the single (precessional) ferromagnetic mode are given by $\omega = \omega _L(1+\alpha ^2)$ and $\tau = (1+\alpha ^2)/(\alpha \omega _L)$, where $\omega _L:=\gamma /\mu _S(2K+4L+\mu _SB+18\kappa )$ is the Larmor frequency. The lifetime of the resonance mode can also be expressed in terms of its frequency via $\tau =1/(\alpha \omega )$.

On ultrashort timescales, where inertial effects can no longer be neglected, a second, nutational mode arises. Analytical calculations of the resonance frequencies and lifetimes of the linearised ILLG equation (for details see methods section) yield

7 $$\begin{aligned} \begin{aligned} \omega _n&= \frac{\text {Im} [\sqrt{\alpha ^2+2i\alpha -4\omega _L\eta -1}] + 1}{2\eta }, \ \tau _n = \frac{2\eta }{\text {Re} [\sqrt{\alpha ^2+2i\alpha -4\omega _L\eta -1}] + \alpha }, \\ \omega _p&= \frac{\text {Im} [\sqrt{\alpha ^2+2i\alpha -4\omega _L\eta -1}] - 1}{2\eta }, \ \tau _p = -\frac{2\eta }{\text {Re} [\sqrt{\alpha ^2+2i\alpha -4\omega _L\eta -1}] - \alpha }. \end{aligned} \end{aligned}$$

Notably, the nutational resonance is shifted by $1/\eta$ relative to the precessional resonance and is thus characterised by a much higher frequency (because the nutational time constant $\eta$ is expected to be much smaller than the precessional period). The nutational mode has a clockwise circular polarisation, while the precessional mode has an anti-clockwise one. This is due to Eq. (5) enforcing a certain polarisation for each resonance mode. This observation was also reported in the literature^5,18^. However, since the expressions in Eq. (7) contain complex square roots, the qualitative dependence of the frequencies and lifetimes on the inertial parameter $\eta$ is not readily apparent.

**Fig. 2:**
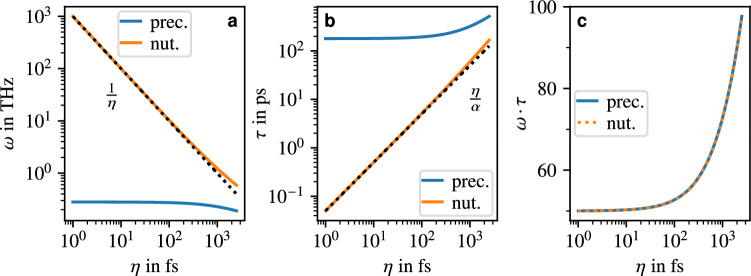
Properties of the precessional and nutational resonance modes in the toy model FM as a function of the inertial parameter $\eta$: (**a**) resonance frequencies, (**b**) lifetimes, and (**c**) effective lifetimes. The black dotted lines indicate the leading-order terms.

The leading orders of the $\eta$ dependence can be obtained through a Taylor expansion. This approach is reasonable here since typically $\alpha \ll 1$ holds for the Gilbert damping, and for the assumed $\eta$ values in the femtosecond range and common values of anisotropy and isotropic exchange, $\omega _L\eta \ll 1$ holds as well. Second-order (one-dimensional) Taylor expansions in $\alpha$ and in $\omega _L\eta$ then yield the following frequencies and lifetimes,

8 $$\begin{aligned} \begin{aligned} \omega _n&\approx \frac{1}{\eta } + \frac{\omega _L}{1+\alpha ^2} \cdot \biggl (1-\omega _L\eta +5\alpha ^2\omega _L\eta \biggr ), \quad \tau _n \approx \frac{\eta \cdot (1+\alpha ^2)}{\alpha } \cdot \frac{1}{1-\omega _L\eta +3\omega _L^2\eta ^2}, \\ \omega _p&\approx \frac{\omega _L}{1+\alpha ^2}\cdot \biggl (1-\omega _L\eta +5\alpha ^2\omega _L\eta \biggr ), \qquad \quad \, \tau _p \approx \frac{1+\alpha ^2}{\omega _L\alpha }\cdot \frac{1}{1 - 3\omega _L\eta }, \end{aligned} \end{aligned}$$

where, for consistency with the precessional resonance frequency and lifetime in the inertia-free case in the limit $\eta \rightarrow 0$, we calculated the Taylor series of the expressions in Eq. (7) expanded with $1+\alpha ^2$. In agreement with the numerical results, which are depicted in Fig. 2, the analytical expressions for the frequencies and lifetimes show that the frequency of the precessional mode decreases with increasing inertial parameter, while its lifetime increases. The leading order expressions for the precessional frequency in lifetime are given by their values in the inertia-free case, while the corrections due to the inertial effects are typically small for $\omega _L\eta \ll 1$. As can be seen from the last terms in the expansions of the frequencies, the inertial effects introduce an additional term containing the Gilbert damping compared to the inertia-free case, which, for typical values of $\eta$, $\omega _L$ and $\alpha$, slightly weakens the decrease in frequency with increasing damping. In the case of $\alpha =0$, our approximations for the resonance frequencies are identical to those obtained by Mondal et al.^18^ and only slightly differ from those by Cherkasskii et al.^19^ and Olive et al.^20^ regarding the coefficients of the higher-order terms in the expansions.

The nutational lifetime is well described by the leading-order expression in Eq. (8), while the other terms account for a small, additional increase in lifetime for large inertial parameters. The approximation for the lifetime of the precessional mode in that limit is, however, much more inaccurate since the expression diverges for $1=3\omega _L\eta$, which may be in the range of possible values of $\omega _L\eta$. Similar to the inertia-free case, the relation $\tau _{n,p}\approx 1/(\alpha \omega _{n,p})$ roughly holds, while $\omega _n\tau _n = \omega _p\tau _p$, as can be seen in Fig. 2(c), which follows from Eq. (7) (see Supplementary Information for the details of the proof). Overall, these last two results establish the connection between the precessional and nutational lifetimes through $\tau _n \approx (\eta \tau _p)/(\eta + \alpha \tau _p)$.

#### Generic antiferromagnet

We now consider a corresponding two-sublattice antiferromagnet with uniaxial second-order on-site anisotropy *K* and isotropic exchange *J* only between spins in opposite sublattices. For this system, the linearised effective field on lattice site *l* in sublattice $X=1,2$ reads

9 $$\begin{aligned} \varvec{H}^l \approx -J \biggl ( \sum _{j_Y} S_x^{j_Y}\hat{\varvec{e}}_x + S_y^{j_Y}\hat{\varvec{e}}_y + S_z^{j_Y}\hat{\varvec{e}}_z \biggr ) + 2KS^l_z \hat{\varvec{e}}_z, \end{aligned}$$

where $j_Y$ are the nearest-neighbours in the opposite sublattice and the exchange constant is defined such that $J>0$.

As for the FM, we will first discuss the resonance frequencies and lifetimes in the absence of inertial effects. We will only discuss the case $B=0$ because, in the inertial case, the linearised system obtained by our methods only allows for analytical solutions in the field-free case. In the inertia-free case, the resonance frequency and lifetime of the doubly degenerate mode are

10 $$\begin{aligned} \omega _\pm = \pm \frac{\sqrt{\tilde{K}^2 + 2\tilde{J}\tilde{K} - \alpha ^2\tilde{J}^2}}{1+\alpha ^2} \qquad \text {and} \qquad \tau = \frac{1+\alpha ^2}{\alpha (\tilde{J}+\tilde{K})}, \end{aligned}$$

where $\tilde{J}:=6\gamma J/\mu _S$ and $\tilde{K}:=2\gamma K/\mu _S$ are the renormalised isotropic exchange and second-order on-site anisotropy, respectively. In contrast to the FM, the frequency and lifetime of the AFM mode depends on the isotropic exchange constant *J*, and, since typically $\tilde{J}\gg \tilde{K}$, the resonance frequency of the AFM is much larger and the lifetime much shorter compared to the FM. This is also known as the exchange enhancement of antiferromagnetic systems. In the case of $\alpha =0$ and $\tilde{J}\gg \tilde{K}$, Eq. (10) becomes $\omega \approx \sqrt{2\tilde{J}\tilde{K}}$, which is consistent with earlier results in the literature^18,21^.

With inertial effects, the eigenvalues corresponding to precessional and nutational modes are each twice degenerate (without external field) and read

11 $$\begin{aligned} \lambda _{p_\pm }&= \frac{1}{6\eta }\cdot \biggl (-3\alpha + E \pm \sqrt{3}\sqrt{6\alpha ^2 - 4A - \frac{C}{D} - D + \frac{18\alpha }{E}}\biggr ), \end{aligned}$$

12 $$\begin{aligned} \lambda _{n_\pm }&= -\frac{1}{6\eta }\cdot \left( 3\alpha + E \pm \sqrt{3}\sqrt{6\alpha ^2 - 4A - \frac{C}{D} - D - \frac{18\alpha }{E}}\right) , \end{aligned}$$

with the abbreviations

$$\begin{aligned} A&= 1 + \alpha ^2 + 2\tilde{J}\eta + 2\tilde{K}\eta , \\ B&= 54(\tilde{J}+\tilde{K})^2\alpha ^2\eta ^2 + 54\tilde{K}(2\tilde{J}+\tilde{K})\alpha ^2\eta ^2 - 18(\tilde{J}+\tilde{K})\alpha ^2\eta A - 36\tilde{K}(2\tilde{J}+\tilde{K})\eta ^2 A + A^3, \\ C&= -12(\tilde{J}+\tilde{K})\alpha ^2\eta + 12\tilde{K}(2\tilde{J}+\tilde{K})\eta ^2 + A^2, \\ D&= \biggl (B + \sqrt{B^2 - C^3}\biggr )^{\frac{1}{3}} \qquad \text {and} \qquad E = \sqrt{3}\sqrt{3\alpha ^2 - 2A + \frac{C}{D} + D}. \end{aligned}$$

Since the matrix corresponding to the above eigenvalues is real, the eigenvalues come in pairs of complex conjugates (the ± indicate the sign of the imaginary parts). For $\alpha =0$ and $\tilde{J}\gg \tilde{K}$ the eigenvalue corresponding to the nutational mode, Eq. (12), can be simplified significantly,

13 $$\begin{aligned} \lambda _{n\pm } \approx \pm \sqrt{-\frac{1}{\eta ^2}-\frac{2\tilde{J}}{\eta }}, \quad \text {i.e.} \quad \omega _{n\pm } \approx \pm \sqrt{\frac{1}{\eta ^2}+\frac{2\tilde{J}}{\eta }}. \end{aligned}$$

**Fig. 3:**
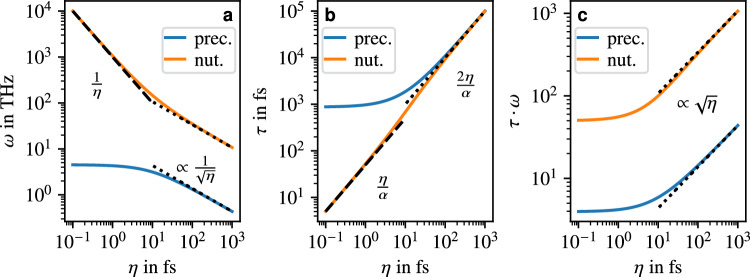
Properties of the precessional and nutational resonance modes for the toy model AFM as a function of the inertial parameter: (**a**) resonance frequencies, (**b**) lifetimes, and (**c**) effective lifetimes. The leading terms are indicated by the dashed lines for small $\eta$ and the dotted lines for large $\eta$. In (**a**), the two dotted lines are $\sqrt{2\tilde{J}/\eta }$ and $\sqrt{\tilde{K}/\eta }$ for nutational and precessional modes, respectively. In (**c**), they are given by $\sqrt{8\tilde{J}\eta }/\alpha$ and $\sqrt{4\tilde{K}\eta }/\alpha$. Because the AFM has two magnetic sublattices, each line represents two degenerate branches.

Thus, in the case of small inertial parameters, the nutational frequency is well approximated by $\omega _{n\pm }\approx \pm 1/\eta$, while in the opposite case $\omega _{n\pm }\approx \pm \sqrt{2\tilde{J}/\eta }$ holds, which is also consistent with the findings of Mondal et al.^18^ and Titov et al.^21^ These same approximations applied to the precessional mode would yield a vanishing precessional frequency. However, assuming $\alpha =0$ but without any assumptions regarding $\tilde{J}$ and $\tilde{K}$, we arrive at

14 $$\begin{aligned} \lambda _{p\pm } = \sqrt{\frac{-1-2\eta (\tilde{J}+\tilde{K})+\sqrt{1+4\eta (\tilde{J}+\tilde{K})+4\tilde{J}^2\eta ^2}}{2\eta ^2}} \end{aligned}$$

in agreement with Titov et al.^21^ In the limit of $\eta \rightarrow 0$ the precessional frequency becomes $\omega _{p\pm }=\pm \sqrt{\tilde{K}(2\tilde{J}+\tilde{K}})$, which is consistent with the resonance frequency in the inertia-free case for $\alpha =0$, cf. Eq. (10). In the limit of large inertial parameters, where the terms in the numerator of lower order in $\eta$ are negligible, the precessional frequency becomes $\omega _{p\pm }\approx \pm \sqrt{\tilde{K}/\eta }$. These approximations for the frequencies and lifetimes in the limits of large and small inertial parameters are also consistent with the numerical results, which are calculated for a non-zero damping parameter and shown in Fig. 3(a).

For the lifetimes, again approximations for the cases of large $\eta$ can be directly obtained from Eqs. (11) and (12), reading

15 $$\begin{aligned} \tau _p = \frac{6\eta }{3\alpha -E} \qquad \text {and} \qquad \tau _n = \frac{6\eta }{3\alpha +E}. \end{aligned}$$

Here, *E* takes the form $E=f(\eta )\cdot \alpha$ with a function $f(\eta )$ that, as numerical calculations show, has the limits $f(\eta )\rightarrow 3$ for $\eta \rightarrow 0$, and $f(\eta )\rightarrow 0$ for $\eta \rightarrow \infty$. Hence, for large $\eta$, the lifetimes of both the precessional and the nutational modes are given by $\tau _{n,p}\approx 2\eta /\alpha$, which fits the numerical results in Fig. 3(b). For $\eta \rightarrow 0$ our numerical calculations show that the precessional lifetime is the same as in the inertia-free case. For the nutational lifetime, Eq. (15) implies $\tau _n\approx \eta /\alpha$, which is verified by the numerical results. Overall, the precessional and nutational lifetimes increase with increasing inertial parameter as shown in Fig. 3(b). Compared to the FM (Fig. 2), the decrease in precessional frequency and increase in precessional lifetime is more drastic for the AFM. Therefore, as suggested by Mondal et al.^18^, antiferromagnets are better suited for the study of inertial dynamics than ferromagnets.

Knowing the frequencies and lifetimes of the precessional and nutational modes in the limits of small and large inertial parameters, the computation of the effective lifetimes in these limits is straightforward. In the case of $\eta \rightarrow \infty$ the effective lifetimes of both precessional and nutational mode is $\tau ^{\text {eff}}_{n,p}\propto \sqrt{\eta }$, while $\tau ^{\text {eff}}_{n}\approx 1/\alpha$ holds for $\eta \rightarrow 0$, both of which can be seen in Fig. 3(c). Furthermore, the effective lifetime of the precessional mode agrees with the lifetime of the antiferromagnetic mode in the inertia-free case.

In the limit of low $\eta$, the inertia only leads to additional high-frequency dynamics without affecting the normal precessional dynamics, such that, in the vein of the Born-Oppenheimer approximation, the two phenomena can be treated separately. In the high-$\eta$ limit, however, a quantitatively correct description of the precessional resonances needs to take the inertial term into account. Which of these limits is realised in nature will depend on the material in question. From a theory perspective, spin inertia is a higher-order relativistic effect, similar to Gilbert damping, and is therefore expected to depend on the strength of spin-orbit coupling in the material^7,8^. One way to determine $\eta$ is by comparison of experimentally observed nutational resonances with $\eta$-dependent theoretical predictions. To this end, we discuss in the following section how spin inertia is predicted to manifest in real antiferromagnetic materials depending on the value of $\eta$.

### Nutation in material-specific models for ${\hbox {SmErFeO}_{3}}$ and ${\hbox {Fe}_{2}\hbox {O}_{3}}$

${\hbox {SmErFeO}_3}$ is a canted four-sublattice AFM with a Pnam orthorhombic structure^22^, for which, in the low-temperature phase, the spins are oriented approximately along the *c*-axis. The canting of the spins, originating from an oxygen-mediated DMI, gives rise to a net magnetisation parallel to the *b*-axis in the ground state, which is small compared to the magnetisation of each sublattice. Using the parameters presented by Weiss et al. ^14^, we only model the magnetic moments on the Fe sites of the orthoferrite (since the rare earth moments only order for temperatures below about 6K^14^).

Hematite is an insulating AFM that crystallises in the corundum structure^15^ with a rhombohedral primitive unit cell, whose diagonal is parallel to the crystal symmetry axis, along which the spins are oriented at low temperatures. In this low temperature phase, hematite is a perfect antiferromagnet. The parameters of the hematite model are the ones obtained by Dannegger et al. ^15^.

The Gilbert damping parameter is known to be rather low in both hematite and orthoferrites resulting in large spin-transport distances ^23–25^. The precise values vary depending on the quality of the sample. Here we use $\alpha = {10^{-4}}$ for hematite and $\alpha = {10^{-3}}$ for the orthoferrite, which is comparable to values that can be found elsewhere in literature ^14,26^ but errs slightly on the side of larger damping.

#### Precessional and nutational resonances

For these complex four-sublattices materials, analytically solving the linearised ILLG equation is no longer possible since it would require the calculation of the eigenvalues of a $16\times 16$ matrix. However, we can still compute these eigenvalues, and thus the resonance frequencies and lifetimes, numerically. Because these materials have four magnetic sublattices per unit cell, they have four distinct precessional resonance modes (although some of them can be degenerate). Two of them are at low frequencies, which are (similar to a two-sublattice antiferromagnet) determined by the product of anisotropy and exchange energy. In these modes, the spins remain almost (but not entirely) parallel. The other two, by contrast, are modes where spins within a single unit cell are strongly canted. These are at very high frequencies (similar to excitations at the Brillouin zone edge), and are dominated by the exchange energy between the canted sublattices, which is why they are sometimes called exchange modes.

**Fig. 4:**
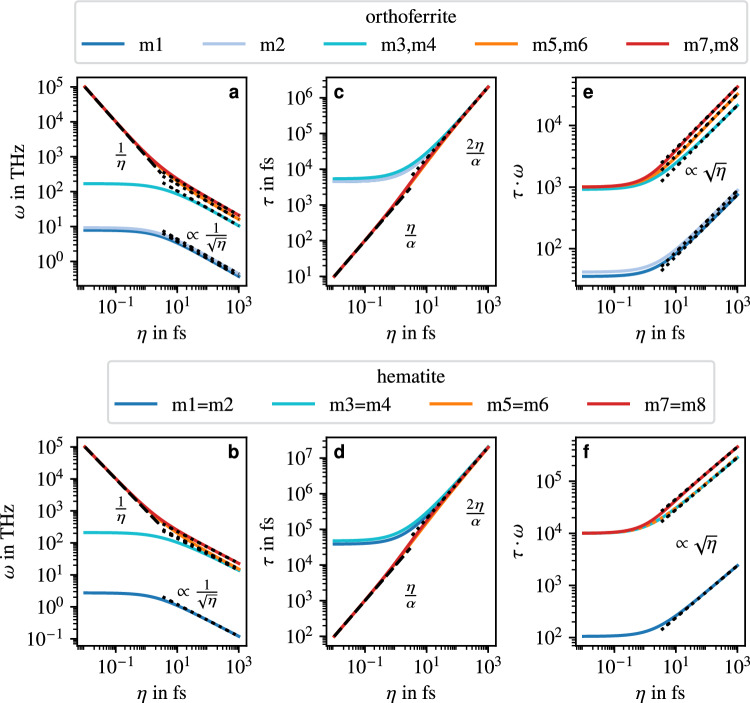
Properties of precessional (blue, turquoise) and nutational (orange, red) resonance modes for the orthoferrite and hematite: (**a, b**) resonance frequencies, (**c, d**) lifetimes, and (**e, f**) effective lifetimes. In the orthoferrite, the higher-frequency modes (m3 to m8) come in pairs that are very close together but still distinct. In hematite, by contrast, all modes come in two exactly degenerate pairs. Again, leading terms are indicated by the dashed and dotted lines (for small and large $\eta$, respectively).

By the same token, there are also four nutational resonance modes. The computed precession and nutation frequencies of the orthoferrite and hematite as a function of the inertial parameter $\eta$ are shown in Fig. 4(a–b).

Analogous to the AFM, the resonance frequencies of these materials are strongly decreasing with increasing inertial parameter $\eta$. The relative frequency gap between the lower-frequency precessional modes (m1 and m2) and the nutational modes (m5 to m8) is roughly the same as for the toy model AFM. For inertial parameters in the order of 10fs or larger, the frequencies of the high-frequency precessional modes (exchange modes, m3 and m4) are similar to those of the nutational modes.

Comparing the orthoferrite and hematite qualitatively, the $\eta$ dependence of the resonance frequencies are quite similar. Apart from the higher numerical values of the hematite frequencies, the main difference between the two systems is that for hematite the relative frequency gap between the precessional and nutational modes is slightly smaller. The close resemblance to the toy model AFM is also evident from the fact that, for the nutational frequencies of the orthoferrite and hematite, the same analytical results as in the case of the toy model AFM can be found in the limit of small inertial parameters, namely, $\omega _n\approx 1/\eta$. This also applies for large inertial parameters (greater than about 100fs), where we find $\omega _{n,p}\propto 1/\sqrt{\eta }$, analogous to the toy model AFM.

For hematite, measurements show that the lower-frequency precessional resonance lies at about 200GHz close to zero temperature^27–30^. In Fig. 4(b) this corresponds to an inertial parameter of a few hundred femtoseconds. At $\eta =0$ our model predicts a resonance frequency that is about a factor of ten larger than the experimental value of about 200GHz. However, inertial effects are not solely responsible for this discrepancy as, close to zero temperature, quantum effects might play a significant role which are neglected in our model. Therefore, the order of magnitude of the inertial parameter in hematite is likely a bit smaller than the few hundred femtoseconds suggested by Fig. 4. The resonance frequency of the higher precessional modes near the Morin transition is reported to be roughly 150THz^31^, which, assuming the mode is roughly temperature-independent, would correspond to an inertial parameter of only a few femtoseconds.

#### Precessional and nutational lifetimes

The $\eta$ dependence of the lifetimes of both materials is shown in Fig. 4(c–d). Analogous to the resonance frequencies, the lifetimes are also strongly affected by the inertial parameter. In particular, the precessional lifetimes also strongly increase with $\eta$ and are essentially identical to the nutational lifetimes for inertial parameters of about 10fs or larger. Again, no qualitative difference between the two materials can be observed. Moreover, the orthoferrite and hematite also behave in the same way as the toy model AFM in terms of the $\eta$ dependence of the lifetimes of the resonance modes. In the limits of large ($\eta >{100}{fs}$) and small ($\eta <{1}{fs}$) inertial parameters, the expressions for the lifetimes of the resonance modes are analogous to those of the AFM, that is, in the former case $\tau _{n,p}\approx 2\eta /\alpha$ and in the latter case $\tau _n\approx \eta /\alpha$.

Despite of the larger frequency gap (in particular between modes m1 and m2 and the nutational modes), the lifetimes of the different precessional modes of each material are almost identical for large inertial parameters. Consequently, the effective lifetimes, i.e. the products $\omega \cdot \tau$, of the lower-frequency precessional modes are much shorter than those of the other modes. On the other hand, for large inertial parameters, the frequencies and lifetimes of the higher-frequency precessional modes are very similar to those of the nutational modes and consequently their effective lifetimes are almost identical.

In detail, the dependence of the numerically computed effective lifetimes of the resonance modes on the inertial parameter are depicted in Fig. 4(e–f). The effective lifetimes of the lower-frequency precessional modes and those of the nutational modes differ by about two orders of magnitude in the case of hematite and slightly less in the case of the orthoferrite, which therefore is about the same difference as the precessional and nutational modes of the toy model AFM have.

Generally, the dependence of the effective lifetimes on the inertial parameter follow those of the toy model AFM closely and are thus also very similar to one another. However, it is noteworthy that, while also being quite similar for the orthoferrite, the effective lifetime of the higher-frequency precessional modes and the lower-frequency nutational modes in hematite are effectively identical. A similar behaviour could also be observed for the modes of the toy model FM in Fig. 2.

As for the frequencies and lifetimes, the similarities of the orthoferrite and hematite to the toy model AFM are also reflected quantitatively in the expressions for the effective lifetimes $\tau _{n,p}^{\text {eff}}\propto \sqrt{\eta }$ and $\tau _n^{\text {eff}}\approx 1/\alpha$ for small and large inertial parameter, respectively, which are exactly the same as for the toy model AFM.

#### Dynamics of nutational resonance modes

**Fig. 5:**
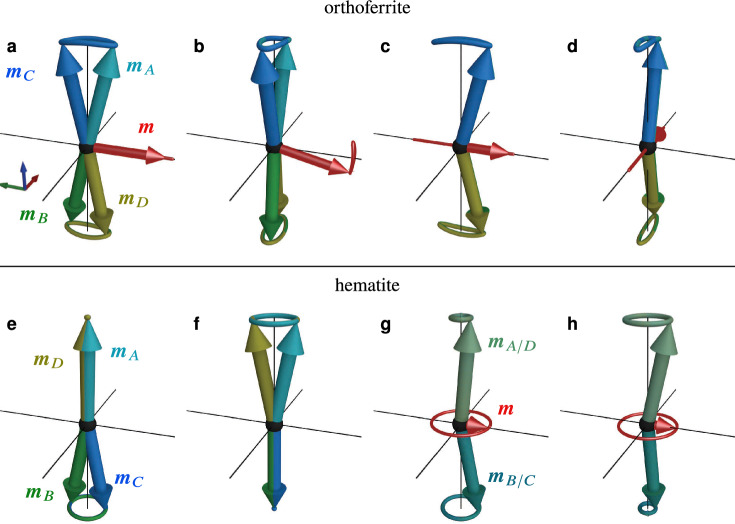
Dynamics of the sublattice magnetisation vectors $\varvec{m}_{A,B,C,D}$ of the nutational resonance modes (m5 to m8) for the orthoferrite (**a–d**) and hematite (**e–h**) model, sorted by frequency in ascending order. The net magnetisation vector $\varvec{m}$ is amplified by a factor of 250 in (**a**) and (**b**) and by a factor of two in the other plots (in ( **e–f**), the total magnetisation vanishes).

For the orthoferrite, the net magnetisation of the resonance modes can be decomposed into a constant part, which is due to the spin canting in the ground state, and a dynamical part. The lower-frequency nutation modes of the orthoferrite depicted in Fig. 5(a–b) resemble its (precessional) exchange modes closely^14^, we will thus call them nutational exchange modes. Compared to the precessional exchange modes, the polarisation of the nutational exchange modes is opposite, similar to the case of the FM.

For these nutational exchange modes, the dynamic component of the net magnetisation is small compared to the static net magnetisation caused by the canted ground state and leads to oscillations either along the *b*-axis or in the *ac*-plane. Qualitatively, the same behaviour can also be observed for the precessional modes^14^.

In the higher-frequency nutational modes, Fig. 5(c–d), the opposite magnetic sublattices are significantly canted, which leads to a dynamical net magnetisation comparable in magnitude to the saturation moment and much larger than the weak ferromagnetic moment caused by the canted ground state. In this respect, the two higher-frequency nutational modes of the orthoferrite are similar to the nutational modes of the toy model AFM, the main difference being that the trajectories in *ab*-plane are elliptical instead of circular due to the biaxial anisotropy of the orthoferrite. In particular the highest-frequency mode, where the resulting net magnetisation oscillates along the *a*-axis, is very distinct from all the precessional modes, which may be used to discern it experimentally.

Hematite, on the other hand, has a uniaxial anisotropy and therefore symmetric modes in its low-temperature phase. The eigenfrequencies are hence doubly degenerate with two-dimensional eigenspaces. This means that the (normalised) basis for this subspace, i.e. the eigenmodes of the system, are ambiguous. In Fig. 5(e–f), the set of basis modes was chosen such that each mode is symmetric in the basal plane. Strikingly, the lower-frequency nutational resonance modes have zero net magnetisation. This situation is similar to the lower-frequency nutational modes in the orthoferrite, where the magnetisation of the resonance modes is no larger than the weak ferromagnetic moment caused by the spin canting. As with the orthoferrite, the polarisation these modes is also opposite compared to the precessional modes. The higher-frequency nutational modes in hematite, see Fig. 5(g–h), are again similar to the nutational modes of the toy model AFM, with a large net magnetisation caused by the two sublattices precessing with different amplitudes.

### Comparison

In terms of their dependence on the inertial parameter, the resonance modes of the orthoferrite and hematite behave very similar to the toy model AFM. However, we also find similarities to the resonance modes of the toy model FM, namely, the effective lifetime of the precessional and nutational exchange modes being almost (in the case of the orthoferrite) or exactly (in the case of hematite) identical. This motivates a more quantitative comparison of the two materials. Since frequencies and damping in the two materials are very different this requires some sort of normalisation process. In the following, we will therefore study the dependence of the normalised frequencies on an inertial parameter, which is for each mode normalised to the mode’s period duration in the inertia-free case.

**Fig. 6:**
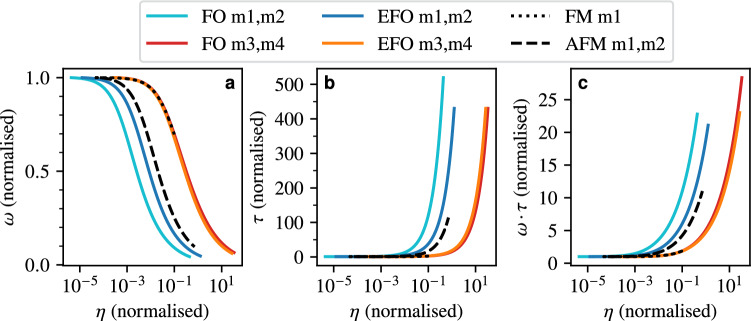
Comparison of the precessional modes of the orthoferrite (EFO) and hematite (FO) to the resonance modes of the toy-model FM and AFM. In (**a**), for each resonance mode, the frequency normalised to its value in the inertia-free case is plotted versus the inertial parameter normalised to the period duration of that mode in the inertia-free case. Figures (**b**) and (**c**) show the same for the lifetimes and effective lifetimes.

Fig. 6(a) shows that the frequencies of the lower-frequency modes (m1 and m2) of the orthoferrite (EFO) and hematite (FO) decrease significantly stronger than those of the higher-frequency modes (m3 and m4) for increasing inertial parameter.

Therefore, the lower-frequency modes behave rather similar to the precessional mode of the toy model AFM, while the exchange modes behave similarly to the precessional mode of the toy model FM in that the decrease in frequency is much weaker. Similarly, as depicted in Fig. 6(b), the lifetimes of the lower-frequency modes increase more strongly than those of the exchange modes. Thus, analogous to the frequencies, the lower-frequency modes behave more like the AFM modes, while the exchange modes behave more like the FM mode. As shown in Fig. 6(c) this is also true for the effective lifetimes.

This furthermore allows for a classification of the nutational modes as either more FM-like or more AFM-like modes in terms of how they are affected by inertia. For the classification we can also utilise the earlier observation that the effective lifetimes of the precessional and nutational exchange modes are basically identical for the orthoferrite as well as hematite. This was also an important property of the precessional and nutational mode of the toy model FM. Thus, we conclude that the nutational exchange modes correspond to the precessional exchange modes. Consequently, we can then also classify the nutational exchange modes as FM-like modes.

Of course, all nutational resonance modes display a strong canting between sublattices and could therefore be described as exchange modes. But there is a particular similarity between the lower-frequency nutational resonances and the precessional exchange modes in that there is a large canting induced between spins that are parallel in the ground state, and this canting is symmetric in such a way that the effect on the net magnetisation is minimal (in the case of the orthoferrite) or vanishes entirely (in the case of hematite).

By contrast, the higher-frequency nutational resonances display a strong canting between *anti*parallel spins, resulting in a large net magnetisation, which is similar to the nutational resonance of the two-sublattice toy model AFM. Therefore, we may classify these higher-frequency nutational modes as AFM-like modes, which correspond to the lower-frequency precessional modes.

## Conclusion

We have presented analytical and numerical calculations of the frequencies, lifetimes, and magnetisation dynamics of nutational resonance modes in antiferromagnets. We have shown how the nutational resonance dynamics differ from the known precessional resonance modes, both in simple toy models and in real-life materials that are of interest in current spintronics research. The nutational resonances we calculated are characterised by a canting between the magnetic sublattices, similar to high-energy precessional modes. The resulting net magnetisation exhibits a complex and material-dependent behaviour that is in some cases qualitatively different from the corresponding precessional dynamics. These differences may be used to identify nutational resonances and differentiate them from their precessional counterparts.

We have furthermore analysed the systematic differences between ferro- and antiferromagnetic nutation and provided analytical expressions for the asymptotic behaviour of the resonance frequencies and lifetimes. In the more complex four-sublattice antiferromagnets, we found similar characteristics between the high-frequency precessional exchange modes and the lower-frequency nutational resonance modes, namely canting between parallel spins with minimal or no effect on the total magnetisation. On the other hand, the low-frequency precessional resonances and the higher-frequency nutational modes reflect the behaviour observed with the two-sublattice antiferromagnet, namely canting between antiparallel sublattices and a large net magnetisation associated with the nutational resonance.

In thermal equilibrium, nutational resonance modes would be excited less than the precessional modes because of their higher energy. But we have seen that, for sufficiently large nutational parameters, the difference to the precessional exchange modes could become small and they might end up in similar orders of magnitude. Furthermore, we have calculated that the highest-frequency nutational resonance modes show a remarkably strong amplitude in the *net* magnetisation, which could even make them the dominant signal in a measurement sensitive to the total magnetisation. The aforementioned qualitative differences together with the expected frequency ranges in comparison to precessional resonances can be used to look for experimental signatures of inertial spin dynamics. Such direct observations in a variety of different materials would significantly expand our understanding of the role of inertia in spin dynamics, thereby testing the limits of the validity of the conventional LLG equation.

## Methods

### Linearisation of the ILLG in the case of an arbitrary ground state

In this section, we present the linearisation of the ILLG equation for systems with arbitrary commensurate ground states. Starting from the Hamiltonian

16 $$\begin{aligned} \mathcal {H} = -\frac{1}{2}\sum _{i,j} (\varvec{S}^i)^T\varvec{\mathfrak {J}}^{ij}\varvec{S}^j - \sum _i (\varvec{S}^i)^T\varvec{\mathfrak {K}}^i\varvec{S}^i - \sum _i \sum _{\beta ,\eta ,\kappa ,\lambda } \varvec{\mathfrak {L}}^i_{\beta \eta \kappa \lambda } S^i_\beta S^i_\eta S^i_\kappa S^i_\lambda - \sum _i \mu ^i_S\varvec{B}\cdot \varvec{S}^i, \end{aligned}$$

and the explicit formulation of the ILLG equation,

17 $$\begin{aligned} \frac{\mathrm d^2\varvec{S}^l}{\mathrm dt^2} = -\frac{\gamma }{\mu _S^l\eta }\varvec{S}^l\times \left( \varvec{S}^l\times \varvec{H}^l\right) - \frac{\alpha }{\eta }\frac{\mathrm d\varvec{S}^l}{\mathrm dt} - \frac{1}{\eta }\varvec{S}^l\times \frac{\mathrm d\varvec{S}^l}{\mathrm dt} - \varvec{S}^l\left( \frac{\mathrm d\varvec{S}^l}{\mathrm dt}\right) ^2, \end{aligned}$$

we derive a homogeneous linear system of differential equations, describing the time evolution of the collective sublattice magnetisations.

**Collinear Ground State** First, we assume a collinear ground state along the *z*-axis, i.e. $|S^l_x|,|S^l_y|\ll |S^l_z|\approx 1$, as well as $\dot{S}^l_z\approx 0$. The effective field of the *l*-th spin in sublattice *X* (strictly speaking, *X* corresponds to one element of the magnetic basis of the system), $\varvec{H}^l=-\frac{\mathrm d\mathcal {H}}{\mathrm d\varvec{S}^l}$, can then be linearised with respect to $S_x$ and $S_y$, which yields

18 $$\begin{aligned} {^X\!}\varvec{H}^l \approx&\sum _j \varvec{\mathfrak {J}}^{lj}\varvec{S}^j + 2{^X\!}\varvec{\mathfrak {K}}\varvec{S}^l + \sum _\lambda \begin{pmatrix} {^X\!}\bar{\varvec{\mathfrak {L}}}_{x\lambda }(S^l_z)^2S^l_\lambda \\ {^X\!}\bar{\varvec{\mathfrak {L}}}_{y\lambda }(S^l_z)^2S^l_\lambda \\ {^X\!}\bar{\varvec{\mathfrak {L}}}_{z\lambda }(S^l_z)^2S^l_\lambda \end{pmatrix} + \mu _S^l\varvec{B}. \end{aligned}$$

Here, we assumed that the anisotropy at lattice site *l* only depends on the sublattice *X* to which the site *l* belongs. Similarly, we also assume that the *z*-component of the spins is independent of the lattice site *l* and again only on the sublattice containing the site *l*, i.e. $S^l_z\equiv {^X\!}S_z \in \{-1,+1\}$. This is justified by the earlier assumptions $S^l_z\approx 1$ and $\dot{S}^l_z\approx 0$. Substituting this linearised effective field into the explicit ILLG equation (2), and, then linearizing the result with respect to $S_x$, $S_y$ and their respective velocities produces the following first-order coupled differential equations for the dynamics of the spins:

19 $$\begin{aligned} \frac{\mathrm d}{\mathrm dt} {^X\!}S_x^l&= {^X\!}\dot{S}_x^l \end{aligned}$$

20 $$\begin{aligned} \frac{\mathrm d}{\mathrm dt} {^X\!}S_y^l&= {^X\!}\dot{S}_y^l \end{aligned}$$

21 $$\begin{aligned} \frac{\mathrm d}{\mathrm dt} {^X\!}\dot{S}_x^l&= \frac{ -{^X\!}\gamma }{ {^X\!}\mu _s {^X\!}\eta } \Bigg \{ \left( {^X\!}A_{xx} + {^X\!}B_{xx} \right) {^X\!}S_x^l + \left( {^X\!}A_{xy} \right) {^X\!}S_y^l \nonumber \\&\quad \, + \sum _Y \sum _{j_Y} C^{lj_Y}_{xx} {^Y\!}S_x^{j_Y} + \sum _Y \sum _{j_Y} C^{lj_Y}_{xy} {^Y\!}S_y^{j_Y} + {^X\!}f_x \Bigg \} \nonumber \\&\quad \, + \left( -\frac{{^X\!}\alpha }{{^X\!}\eta } \right) {^X\!}\dot{S}_x^l + \left( \frac{{^X\!}S_z}{{^X\!}\eta }\right) {^X\!}\dot{S}_y^l \end{aligned}$$

22 $$\begin{aligned} \frac{\mathrm d}{\mathrm dt} {^X\!}\dot{S}_y^l&= \frac{ -{^X\!}\gamma }{ {^X\!}\mu _s {^X\!}\eta } \Bigg \{ \left( {^X\!}A_{yx} \right) {^X\!}S_x^l + \left( {^X\!}A_{yy} + {^X\!}B_{yy} \right) {^X\!}S_y^l \nonumber \\&\quad \, + \sum _Y \sum _{j_Y} C^{lj_Y}_{yx} {^Y\!}S_x^{j_Y} + \sum _Y \sum _{j_Y} C^{lj_Y}_{yy} {^Y\!}S_y^{j_Y} + {^X\!}f_y \Bigg \} \nonumber \\&\quad \, + \left( -\frac{{^X\!}S_z}{{^X\!}\eta } \right) {^X\!}\dot{S}_x^l + \left( -\frac{{^X\!}\alpha }{{^X\!}\eta }\right) {^X\!}\dot{S}_y^l \end{aligned}$$

Here we used the following $2\times 2$ coefficient matrices:

$$\begin{aligned} {^X\!}A_{xx}&:= 2 \left( {^X\!}\mathfrak {K}_{zz} - {^X\!}\mathfrak {K}_{xx} \right) + \left( {^X\!}\bar{\mathfrak {L}}_{zz} - {^X\!}\bar{\mathfrak {L}}_{xx} \right) + {^X\!}S_z {^X\!}\mu _s B_z \\ {^X\!}A_{xy}&:= -2{^X\!}\mathfrak {K}_{xy} -{^X\!}\bar{\mathfrak {L}}_{xy} \\ {^X\!}A_{yx}&:= -\left( 2 {^X\!}\mathfrak {K}_{yx} + {^X\!}\bar{\mathfrak {L}}_{yx} \right) \\ {^X\!}A_{yy}&:= 2 \left( {^X\!}\mathfrak {K}_{zz} - {^X\!}\mathfrak {K}_{yy} \right) + \left( {^X\!}\bar{\mathfrak {L}}_{zz} - {^X\!}\bar{\mathfrak {L}}_{yy} \right) + {^X\!}S_z {^X\!}\mu _s B_z \end{aligned}$$

$$\begin{aligned} {^{X}\!}B_{xx}&:= \sum _Y \sum _{j_Y} \mathfrak {J}^{lj_Y}_{zz} {^X\!}S_z {^Y\!}S_z,&C^{l,j_Y}_{xx}&:= - \mathfrak {J}^{lj_Y}_{xx} \\ {^{X}\!}B_{xy}&:= 0,&C^{l,j_Y}_{xy}&:= - \mathfrak {J}^{lj_Y}_{xy} \\ {^{X}\!}B_{yx}&:= 0,&C^{l,j_Y}_{yx}&:= - \mathfrak {J}^{lj_Y}_{yx} \\ {^{X}\!}B_{yy}&:= \sum _Y \sum _{j_Y} \mathfrak {J}^{lj_Y}_{zz} {^X\!}S_z {^Y\!}S_z,&C^{l,j_Y}_{yy}&:= - \mathfrak {J}^{lj_Y}_{yy} \end{aligned}$$

and the the residual term ${^X\!}\varvec{f}$ given by

23 $$\begin{aligned} {^X\!}f_{x}&:= -\left( \sum _Y \sum _{j_Y} \mathfrak {J}^{lj_Y}_{xz} \, {^Y\!}S_z\right) - 2\cdot {^X\!}S_z {^X\!}\mathfrak {K}_{xz} - {^X\!}S_z {^X\!}\bar{\mathfrak {L}}_{xz} - {^X\!}\mu _S {^X\!}B_x, \end{aligned}$$

24 $$\begin{aligned} {^X\!}f_{y}&:= -\left( \sum _Y \sum _{j_Y} \mathfrak {J}^{lj_Y}_{yz} \, {^Y\!}S_z\right) - 2\cdot {^X\!}S_z {^X\!}\mathfrak {K}_{yz} - {^X\!}S_z {^X\!}\bar{\mathfrak {L}}_{yz} - {^X\!}\mu _S {^X\!}B_y, \end{aligned}$$

which is constant with respect the *x*- and *y*-coordinates of the spins and their velocities. Note that because of the summation over $j_Y$ and all sublattices *Y* as well as the assumption of an infinite lattice, the coefficient matrix ${^{X}\!}B$ and the residual term ${^{X}\!}\varvec{f}$ are independent of the lattice sites *l* and $j_Y$ and only depend on the sublattice *X*. Furthermore, we employed ${^X\!}S_z=\pm 1$, that is ${^X\!}S_z^2=1$.

Solving this system still requires solving four coupled differential equations per spin. Since we are only interested in the homogeneous case (with $\varvec{k} = 0$), we average the spins over the entire sublattice *X*:

25 $$\begin{aligned} {^X\!}\hat{S}_\beta = \frac{1}{N}\sum _n {^X\!}S_\beta ^n, \qquad \beta \in \{x,y\}, \end{aligned}$$

where *N* is the number of spins per sublattice, which is independent of the sublattice *X*. This ultimately leaves us with the system

26 $$\begin{aligned} \frac{\mathrm d}{\mathrm dt} {^X\!}\hat{S}_x&= {^X\!}\dot{\hat{S}}_x, \end{aligned}$$

27 $$\begin{aligned} \frac{\mathrm d}{\mathrm dt} {^X\!}\hat{S}_y&= {^X\!}\dot{\hat{S}}_y, \end{aligned}$$

28 $$\begin{aligned} \frac{\mathrm d}{\mathrm dt} {^X\!}\dot{\hat{S}}_x&= \frac{ -{^X\!}\gamma }{ {^X\!}\mu _s {^X\!}\eta } \Bigg \{ \left( {^X\!}A_{x x} + {^X\!}B_{x x} \right) {^X\!}\hat{S}_x + \left( {^X\!}A_{x y} \right) {^X\!}\hat{S}_y \nonumber \\&\quad + \sum _Y {^{XY}\!}\hat{C}_{x x} {^Y\!}\hat{S}_x + \sum _Y {^{XY}\!}\hat{C}_{x y} {^Y\!}\hat{S}_y + {^X\!}\hat{f}_x \Bigg \} -\frac{{^X\!}\alpha }{{^X\!}\eta } {^X\!}\dot{\hat{S}}_x + \frac{{^X\!}S_z}{{^X\!}\eta } {^X\!}\dot{\hat{S}}_y, \end{aligned}$$

29 $$\begin{aligned} \frac{\mathrm d}{\mathrm dt} {^X\!}\dot{\hat{S}}_y&= \frac{ -{^X\!}\gamma }{ {^X\!}\mu _s {^X\!}\eta } \Bigg \{ \left( {^X\!}A_{y x} \right) {^X\!}\hat{S}_x + \left( {^X\!}A_{y y} + {^X\!}B_{y y} \right) {^X\!}\hat{S}_y \nonumber \\&\quad + \sum _Y {^{XY}\!}\hat{C}_{y x} {^Y\!}\hat{S}_x + \sum _Y {^{XY}\!}\hat{C}_{y y} {^Y\!}\hat{S}_y + {^X\!}\hat{f}_y \Bigg \} -\frac{{^X\!}S_z}{{^X\!}\eta } {^X\!}\dot{\hat{S}}_x -\frac{{^X\!}\alpha }{{^X\!}\eta } {^X\!}\dot{\hat{S}}_y, \end{aligned}$$

where the coefficient matrices ${^{XY}\!}\hat{C}$ are defined by

30 $$\begin{aligned} {^{XY}\!}\hat{C}_{\beta \delta } := \sum _{l} C_{\beta \delta }^{lj_Y}, \qquad \beta ,\delta \in \{x,y\}. \end{aligned}$$

The ${^{XY}\!}\hat{C}$ are independent of *l* and $j_Y$ due to the assumption of an infinite lattice.

For a finite ${^{X}\!}\varvec{\hat{f}}$, the linear system would be non-homogeneous, but in the ground state, where all spins are parallel to the *z*-axis and the velocities are zero, there should not be a torque action on the spins, and thus $\hat{f}$ must vanish. Otherwise, the system would be driven out of the supposed ground state. Therefore, the resulting linear system of ordinary differential equations must be homogeneous and has the form

31 $$\begin{aligned} \frac{\mathrm d}{\mathrm dt} \hat{\mathcal {S}} = M \hat{\mathcal {S}}, \qquad \hat{\mathcal {S}} := \left( {^\textrm{A}\!}\hat{S}_x, {^\textrm{A}\!}\hat{S}_y, {^\textrm{A}\!}\dot{\hat{S}}_x, {^\textrm{A}\!}\dot{\hat{S}}_y, {^\textrm{B}\!}\hat{S}_x, \dots \right) ^T \end{aligned}$$

where $\hat{\mathcal {S}}$ contains the deflections in *x*- and *y*-direction relative to the ground state spin orientations and the velocities of these spins for each sublattice. The coefficient matrix *M* in Eq. (31) is given by

32 $$\begin{aligned} M&:= \begin{pmatrix} \varvec{0} & I & \varvec{0} & \varvec{0} & \ldots \\ {^A\!}A + {^A\!}B & {^A\!}D & \varvec{0} & \varvec{0} & \ldots \\ \varvec{0} & \varvec{0} & \varvec{0} & I & \\ \varvec{0} & \varvec{0} & {^B\!}A + {^B\!}B & {^B\!}D & \\ \vdots & \vdots & & & \ddots \end{pmatrix} + \begin{pmatrix} \varvec{0} & \varvec{0} & \varvec{0} & \varvec{0} & \ldots \\ {^{AA}\!}\hat{C} & \varvec{0} & {^{AB}\!}\hat{C} & \varvec{0} & \\ \varvec{0} & \varvec{0} & \varvec{0} & \varvec{0} & \ldots \\ {^{BA}\!}\hat{C} & \varvec{0} & {^{BB}\!}\hat{C} & \varvec{0} & \\ & \vdots & & \vdots & \ddots \end{pmatrix}, \end{aligned}$$

with the $2\times 2$ identity matrix *I*, the $2\times 2$ zero matrix $\varvec{0}$ and the matrix

33 $$\begin{aligned} {^X\!}D := \frac{1}{{^X\!}\eta }\begin{pmatrix} -{^X\!}\alpha & {^X\!}S_z \\ -{^X\!}S_z & -{^X\!}\alpha \\ \end{pmatrix}, \end{aligned}$$

containing the Gilbert damping parameter, the inertial parameter and ground state spin orientation of sublattice *X*.

**Procedure in the case of a non-collinear ground state** In the case of a non-collinear ground state, the system can simply be transformed into a system with a collinear ground state by rotating each sublattice *X* separately using the matrices ${^X\!}\mathfrak {R}$ defined by

34 $$\begin{aligned} {^X\!}\tilde{\varvec{S}}_0 := {^X\!}\mathfrak {R} {^X\!}\varvec{S}_0 = \pm \hat{\varvec{e}}_z, \end{aligned}$$

where ${^X\!}\varvec{S}_0$ is the original ground state orientation of the spins in sublattice *X*. Under this transformation the Hamiltonian remains invariant, the effective field transforms like the spins and the ILLG equation stays invariant. Thus, the same procedure as for the collinear system can be employed for the rotated system to calculate its eigenvalues, i.e. resonance frequencies and lifetimes, and its eigenvectors. The latter are then transformed back via

35 $$\begin{aligned} {^X\!}\varvec{S} = {^X\!}\mathfrak {R}^T\, {^X\!}\tilde{\varvec{S}}. \end{aligned}$$

Independently, a similar approach to linearising the ILLG equation for non-collinear systems was derived and presented in Ref. ^32^.

### Toy model parametrisation

The values shown in Fig. 2 and 3 for the FM and AFM toy models use the parameters $\alpha =0.02$, $\gamma ={1.76\times 10^{11}}{\textrm{rad}\,\textrm{s}^{-1}\textrm{T}^{-1}}$, $K={10^{-23}}{\textrm{J}}$, $J={10^{-21}}{\textrm{J}}$, and $\mu _S=2\mu _B$.

## Supplementary Information

Supplementary Information.

## Data Availability

The data presented in this work is available from the corresponding author upon reasonable request.
