# Supplementary material for: Nutational resonance modes in antiferromagnetic materials
Source: Sci Rep. 2025 Jul 1;15:21543. doi: 10.1038/s41598-025-08746-0 (PMC12214598; doi:10.1038/s41598-025-08746-0)
Supplement: Supplementary file 1 — Supplementary Information. [file 41598_2025_8746_MOESM1_ESM.pdf]

Supplementary Information

Nutational resonance modes in antiferromagnetic materials

David Angster, Tobias Dannegger, Julius Schlegel, Martin Evers, and Ulrich Nowak

Fachbereich Physik, Universität Konstanz, D-78457 Konstanz, Germany

S1 Equality of ferromagnetic precessional and nutational effective life times

The exact solutions for the precessional and nutational frequencies and life times of our toy model ferromagnet are given in Eq. (7) as

$$\omega_n = \frac{\mathrm{Im}[\sqrt{\alpha^2 + 2\mathrm{i}\alpha - 4\omega_L\eta - 1}] + 1}{2\eta}, \qquad \tau_n = \frac{2\eta}{\mathrm{Re}[\sqrt{\alpha^2 + 2\mathrm{i}\alpha - 4\omega_L\eta - 1}] + \alpha},$$
$$\omega_p = \frac{\mathrm{Im}[\sqrt{\alpha^2 + 2\mathrm{i}\alpha - 4\omega_L\eta - 1}] - 1}{2\eta}, \qquad \tau_p = -\frac{2\eta}{\mathrm{Re}[\sqrt{\alpha^2 + 2\mathrm{i}\alpha - 4\omega_L\eta - 1}] - \alpha}.$$

From that, we get the effective life times

$$\omega_n\tau_n = \frac{\mathrm{Im}(z) + 1}{\mathrm{Re}(z) + \alpha} \quad \text{and} \quad \omega_p\tau_p = -\frac{\mathrm{Im}(z) - 1}{\mathrm{Re}(z) - \alpha} \tag{S1}$$
$$\text{where} \quad z := \sqrt{\alpha^2 + 2\mathrm{i}\alpha - 4\omega_L\eta - 1}. \tag{S2}$$

Regardless of which of the two solutions we pick for z, we can write it generally as $z = \varrho e^{\mathrm{i}\varphi}$. Using Euler's formula together with an addition formula, we then get (generally for any complex number):

$$\mathrm{Re}(z)\,\mathrm{Im}(z) = \varrho^2\cos(\varphi)\sin(\varphi) = \tfrac{1}{2}\varrho^2\sin(2\varphi) = \tfrac{1}{2}\,\mathrm{Im}(z^2). \tag{S3}$$

Because here $\mathrm{Im}(z^2) = 2\alpha$, we know that

$$\mathrm{Re}(z)\,\mathrm{Im}(z) = \alpha \quad \Leftrightarrow \quad \mathrm{Re}(z) = \frac{\alpha}{\mathrm{Im}(z)}. \tag{S4}$$

Plugging that into equations (S1), we get

$$\omega_n \tau_n = \frac{\mathrm{Im}(z)+1}{\frac{\alpha}{\mathrm{Im}(z)}+\alpha} = \frac{\mathrm{Im}(z)}{\alpha} \cdot \frac{\mathrm{Im}(z)+1}{1+\mathrm{Im}(z)} = \frac{\mathrm{Im}(z)}{\alpha} \tag{S5}$$

and

$$\omega_p \tau_p = -\frac{\mathrm{Im}(z)-1}{\frac{\alpha}{\mathrm{Im}(z)}-\alpha} = \frac{\mathrm{Im}(z)}{\alpha} \cdot \frac{-\mathrm{Im}(z)+1}{1-\mathrm{Im}(z)} = \frac{\mathrm{Im}(z)}{\alpha}. \tag{S6}$$

The effective lifetimes are therefore the same for both modes. □

(The same is not true for the antiferromagnet.)

In the inertia-free case, we have $z = \sqrt{\alpha^2 + 2\mathrm{i}\alpha - 1} = \sqrt{(\alpha + \mathrm{i})^2} = \alpha + \mathrm{i}$, therefore $\mathrm{Im}(z) = 1$ and $\omega\tau = 1/\alpha$. The inertial term leads to an increase of the effective life time in a manner proportional to the reduction of the resonance frequencies:

$$\omega\tau \approx \frac{1}{\alpha}\left(1 + \frac{2\omega_L \eta}{1+\alpha^2} + \mathcal{O}(\eta^2)\right) \tag{S7}$$

S2 Eigenvalues of the antiferromagnetic toy model

In this section, we outline the derivation of the eigenvalues corresponding to resonance frequencies and lifetimes of the toy-model antiferromagnet in the general case, as given in Eqs. (11) and (12) of the main text. The corresponding linear system, obtained as described in the Methods section, reads

$$\frac{\mathrm{d}}{\mathrm{d}t}\begin{pmatrix} {}^{A}\hat{S}_x \\ {}^{A}\hat{S}_y \\ {}^{A}\dot{\hat{S}}_x \\ {}^{A}\dot{\hat{S}}_y \\ {}^{B}\hat{S}_x \\ {}^{B}\hat{S}_y \\ {}^{B}\dot{\hat{S}}_x \\ {}^{B}\dot{\hat{S}}_y \end{pmatrix} = \begin{pmatrix} 0 & 0 & 1 & 0 & 0 & 0 & 0 & 0 \\ 0 & 0 & 0 & 1 & 0 & 0 & 0 & 0 \\ -\frac{\tilde{K}+\tilde{J}}{\eta} & 0 & -\frac{\alpha}{\eta} & \frac{1}{\eta} & -\frac{\tilde{J}}{\eta} & 0 & 0 & 0 \\ 0 & -\frac{\tilde{K}+\tilde{J}}{\eta} & -\frac{1}{\eta} & -\frac{\alpha}{\eta} & 0 & -\frac{\tilde{J}}{\eta} & 0 & 0 \\ 0 & 0 & 0 & 0 & 0 & 0 & 1 & 0 \\ 0 & 0 & 0 & 0 & 0 & 0 & 0 & 1 \\ -\frac{\tilde{J}}{\eta} & 0 & 0 & 0 & -\frac{\tilde{K}+\tilde{J}}{\eta} & 0 & -\frac{\alpha}{\eta} & -\frac{1}{\eta} \\ 0 & -\frac{\tilde{J}}{\eta} & 0 & 0 & 0 & -\frac{\tilde{K}+\tilde{J}}{\eta} & \frac{1}{\eta} & -\frac{\alpha}{\eta} \end{pmatrix}\begin{pmatrix} {}^{A}\hat{S}_x \\ {}^{A}\hat{S}_y \\ {}^{A}\dot{\hat{S}}_x \\ {}^{A}\dot{\hat{S}}_y \\ {}^{B}\hat{S}_x \\ {}^{B}\hat{S}_y \\ {}^{B}\dot{\hat{S}}_x \\ {}^{B}\dot{\hat{S}}_y \end{pmatrix}, \tag{S8}$$

where the abbreviations $\tilde{K} := \frac{\gamma}{\mu_S} 2K$ and $\tilde{J} := \frac{\gamma}{\mu_S} 6J$ are used. The eigenvalues of the coefficient matrix in Eq. (S8) are found as the roots of its characteristic polynomial, which is, in this case, an eight-order polynomial. For such polynomials there are no general analytic expressions for the roots, but due to the symmetry of the matrix in Eq. (S8), after collecting the terms in orders of λ, a binomial formula can be identified and the denominator can be written as the square of a fourth-order polynomial

$$p(\lambda) = \frac{\left[(\tilde{K}^2 + 2\tilde{J}\tilde{K}) + 2\alpha(\tilde{J}+\tilde{K})\lambda + (1+\alpha^2+2\eta(\tilde{J}+\tilde{K}))\lambda^2 + 2\alpha\eta\lambda^3 + \eta^2\lambda^4\right]^2}{\eta^4}. \tag{S9}$$

Therefore, finding its doubly-degenerate roots reduces to solving the quartic equation

$$0 = (\tilde{K}^2 + 2\tilde{J}\tilde{K}) + 2\alpha(\tilde{J} + \tilde{K})\lambda + (1 + \alpha^2 + 2\eta(\tilde{J} + \tilde{K}))\lambda^2 + 2\alpha\eta\lambda^3 + \eta^2\lambda^4, \tag{S10}$$

which can be done analytically. The expressions for these are, however, rather lengthy and after defining some abbreviations take the form given in Eqs. (11) and (12) in the main text.

S3 Low-damping limit of the antiferromagnetic eigenmodes

To derive an expression for the precessional eigenfrequencies and corresponding lifetimes in the case of vanishing damping, as given in Eq. (14) of the main text, we start with the linearised LLG equation for the toy-model antiferromagnet. Inserting $\alpha = 0$ into the linear system, obtained as described in the Methods section, yields

$$\frac{\mathrm{d}}{\mathrm{d}t}\begin{pmatrix} {}^{A}\hat{S}_x \\ {}^{A}\hat{S}_y \\ {}^{A}\dot{\hat{S}}_x \\ {}^{A}\dot{\hat{S}}_y \\ {}^{B}\hat{S}_x \\ {}^{B}\hat{S}_y \\ {}^{B}\dot{\hat{S}}_x \\ {}^{B}\dot{\hat{S}}_y \end{pmatrix} = \begin{pmatrix} 0 & 0 & 1 & 0 & 0 & 0 & 0 & 0 \\ 0 & 0 & 0 & 1 & 0 & 0 & 0 & 0 \\ -\frac{\tilde{K}+\tilde{J}}{\eta} & 0 & 0 & \frac{1}{\eta} & -\frac{\tilde{J}}{\eta} & 0 & 0 & 0 \\ 0 & -\frac{\tilde{K}+\tilde{J}}{\eta} & -\frac{1}{\eta} & 0 & 0 & -\frac{\tilde{J}}{\eta} & 0 & 0 \\ 0 & 0 & 0 & 0 & 0 & 0 & 1 & 0 \\ 0 & 0 & 0 & 0 & 0 & 0 & 0 & 1 \\ -\frac{\tilde{J}}{\eta} & 0 & 0 & 0 & -\frac{\tilde{K}+\tilde{J}}{\eta} & 0 & 0 & -\frac{1}{\eta} \\ 0 & -\frac{\tilde{J}}{\eta} & 0 & 0 & 0 & -\frac{\tilde{K}+\tilde{J}}{\eta} & \frac{1}{\eta} & 0 \end{pmatrix} \begin{pmatrix} {}^{A}\hat{S}_x \\ {}^{A}\hat{S}_y \\ {}^{A}\dot{\hat{S}}_x \\ {}^{A}\dot{\hat{S}}_y \\ {}^{B}\hat{S}_x \\ {}^{B}\hat{S}_y \\ {}^{B}\dot{\hat{S}}_x \\ {}^{B}\dot{\hat{S}}_y \end{pmatrix}, \tag{S11}$$

where the abbreviations $\tilde{K} := \frac{\gamma}{\mu_S} 2K$ and $\tilde{J} := \frac{\gamma}{\mu_S} 6J$ are used. The eigenvalues can be found via the characteristic polynomial of the coefficient matrix in Eq. (S11), which is given by

$$\begin{aligned} p(\lambda) = \frac{1}{\eta^4}\Big[& 4\tilde{J}^2\tilde{K}^2 + 4\tilde{J}\tilde{K}^3 + \tilde{K}^4 \\ & + \lambda^2\Big(4\tilde{J}\tilde{K} + 2\tilde{K}^2 + 12\tilde{J}\tilde{K}^2\eta + 4\tilde{K}^3\eta + 8\tilde{J}^2\tilde{K}\eta\Big) \\ & + \lambda^4\Big(1 + 4\tilde{J}\eta + 4\tilde{K}\eta + 4\tilde{J}^2\eta^2 + 12\tilde{J}\tilde{K}\eta^2 + 6\tilde{K}^2\eta^2\Big) \\ & + \lambda^6\Big(2\eta^2 + 4\tilde{J}\eta^3 + 4\tilde{K}\eta^3\Big) \\ & + \lambda^8\eta^4\Big]. \end{aligned} \tag{S12}$$

This expression can be simplified by factoring out $\left(\tilde{K}^2 + 2\tilde{J}\tilde{K}\right)$ and $\left(1 + 2\eta(\tilde{J} + \tilde{K})\right)$ wherever possible to obtain

$$p(\lambda) = \frac{1}{\eta^4}\Big[(\tilde{K}^2 + 2\tilde{J}\tilde{K})^2 + \lambda^2\Big(2(\tilde{K}^2 + 2\tilde{K}\tilde{J})(1 + 2\eta(\tilde{J} + \tilde{K}))\Big)$$
$$+\lambda^4\Big((1 + 2\eta(\tilde{J} + \tilde{K}))^2 + 2\eta^2(\tilde{K}^2 + 2\tilde{J}\tilde{K})\Big)$$
$$+\lambda^6\Big(2\eta^2(1 + 2\eta(\tilde{J} + \tilde{K}))\Big) + \lambda^8\eta^4\Big], \tag{S13}$$

which is recognised as a binomial formula and can be rewritten as

$$p(\lambda) = \frac{\Big[(\tilde{K}^2 + 2\tilde{J}\tilde{K}) + (1 + 2\eta(\tilde{J} + \tilde{K}))\lambda^2 + \eta^2\lambda^4\Big]^2}{\eta^4}. \tag{S14}$$

This representation shows that the characteristic polynomial has four doubly-degenerate roots, which can be found analytically from the fourth-order polynomial in the square brackets in the numerator via

$$0 = \left(\tilde{K}^2 + 2\tilde{J}\tilde{K}\right) + \left(1 + 2\eta(\tilde{J} + \tilde{K})\right)\lambda^2 + \eta^2\lambda^4. \tag{S15}$$

This can be easily solved by substituting $x := \lambda^2$ to get the second-order polynomial

$$0 = \left(\tilde{K}^2 + 2\tilde{J}\tilde{K}\right) + \left(1 + 2\eta(\tilde{J} + \tilde{K})\right)x + \eta^2 x, \tag{S16}$$

which has the solutions

$$x_\pm = \frac{-1 - 2\eta(\tilde{J} + \tilde{K}) \pm \sqrt{1 + 4\eta(\tilde{J} + \tilde{K}) + 4\eta^2\tilde{J}^2}}{2\eta^2}. \tag{S17}$$

Therefore, the four unique and doubly-degenerate roots of the characteristic polynomial, i.e. the eigenvalues of the system, are given by

$$\lambda_{p\pm,n\pm} = \pm\sqrt{\frac{-1 - 2\eta(\tilde{J} + \tilde{K}) \pm \sqrt{1 + 4\eta(\tilde{J} + \tilde{K}) + 4\eta^2\tilde{J}^2}}{2\eta^2}}, \tag{S18}$$

which is the form given in the main text.
